# Carcinoma-associated fibroblasts derived exosomes modulate breast cancer cell stemness through exonic circHIF1A by miR-580-5p in hypoxic stress

**DOI:** 10.1038/s41420-021-00506-z

**Published:** 2021-06-12

**Authors:** Yanxia Zhan, Junxian Du, Zhihui Min, Li Ma, Wei Zhang, Wei Zhu, Yonglei Liu

**Affiliations:** 1 Department of Hematology, Zhongshan Hospital, Fudan University, Shanghai, China; 2 Research Center, Zhongshan Hospital, Fudan University, Shanghai, China; 3 General Surgery, Zhongshan Hospital, Fudan University, Shanghai, China; 4 Research Center, Zhongshan Hospital Qingpu Branch, Fudan University, Shanghai, China

**Keywords:** Cancer stem cells, Cancer microenvironment

## Abstract

Hypoxia is a common phenomenon in solid tumors. The roles of exosomes from hypoxic breast cancer stroma are less studied. So, the study was aimed to investigate the role of exosomes from hypoxic cancer-associated fibroblasts (CAFs) cells in breast cancer. The circRNA array analysis was performed to screen differential expressed circRNAs between hypoxic and normoxic CAFs exosomes. Candidate circHIF1A (circ_0032138) was screened out and it was confirmed that circHIF1A was up-regulated in the exosomes from hypoxic CAFs and their exosomes. Through investigating cellular functions including cell proliferation and stem cell features, it was demonstrated that hypoxic CAFs exosomes transferred circHIF1A into breast cancer cells, which played an important role in cancer stem cell properties sponging miR-580-5p by regulating CD44 expression. In a summary, circHIF1A from hypoxic CAFs exosomes played an important role in stem cell properties of breast cancer. CircHIF1A may act as a target molecule of breast cancer therapy.

## Introduction

The tumor stroma has a higher ratio in cancer tissues such as breast cancer and influences cancer progression. Carcinoma-associated fibroblasts (CAFs) are the main cell type in tumor stroma and the emerging evidence indicates that CAFs play important roles in cancer progression^1,2^. In tumor stroma, CAFs interact with other cells by direct or undirect interaction^3^. CAFs contribute to cancer stem cell properties by various stresses. CAFs play important roles in reprogramming the tumor microenvironment through the maintenance of the reactive stroma and participate in growth, angiogenesis, immunosuppression, metastasis, metabolism, and stemness^2,3^.

CAFs communicate with other cells like cancer cells, immune cells, adipocytes through secreting various molecules or extracellular vehicles (EVs)^4^. Exosomes are 30–150 nm EVs from diverse cell secretion which exchanges the information between cells^5,6^. Exosomes act as natural vehicles delivering growth factors, chemokines, RNA, noncoding RNAs including microRNAs (miRNAs), long noncoding RNAs (lncRNAs), circular RNA (circRNA), and other soluble mediators to recipient cells^7^. The contents of exosomes from stromal cells could be transferred into breast cancer cells, which lead to changing cancer phenotype^8^.

CircRNAs are rich in exosomes^9,10^. CircRNAs are stable relative to miRNAs because their 5′ cap and 3′ tail are buried in the loop^11^. CircRNA acts as a regulator during carcinogenesis and cancer progression, which is not fully elucidated, but many researchers have stated that circRNAs can function as ceRNAs in tumor biology^9–11^. Exosomes from CAFs exert critical roles in cancer progression via transferring miRNAs, long noncoding RNAs, and circRNAs from one cell to another cell^12,13^. CAFs exosomes delivered miR-181d-5p^14^, miR-34a-5p^15^, lncRNA19^16^, miR-196a^17^ to cancer cells and promoted cancer cell proliferation, metastasis, stemness, and chemoresistance. So, the exosomes from the tumor stroma involve in cancer development and progression. However, the effect of circRNAs from CAFs exosomes on breast cancer cellular functions and pathological mechanisms is still largely unraveled.

In addition, hypoxia is an important phenomenon in the tumor microenvironment^18^. The hypoxia-inducible factor-1α (HIF-1α) is related to noncoding RNAs including circRNAs in cancer^19^. Recently published results showed that the impact of the circRNF20 effect in breast cancer through the miR487/HIF1a/HK2 axis^20^. Interestingly, the hypoxic state affects exosome biogenesis and contents^21^ and involved in aggressive progress in cancer^22–24^. In breast cancer, hypoxia influenced exosome release^25^, however, the roles and molecular mechanisms of circRNAs from CAFs exosomes in breast cancer stemness are not clear.

In this study, we will explore the possible mechanisms of the hypoxic exosomes from tumor stroma on cancer stem cellular phenotypes. The circRNA array was carried out in exosomes from hypoxic tumor stroma to find potential motivators for stem cell properties. It was verified that hypoxic CAFs exosomes circHIF1A played an important role in breast cancer stemness.

## Materials and methods

### Cell lines and culture

Breast cancer cell lines including MDA-MB-231, MDA-MB-468, SKBR3, T47D, BT474, MCF7, and MCF10A which were primarily from ATCC and stored in our lab. Cells were maintained in DMEM/F12, or L15 medium containing 10% fetal bovine serum (FBS), 100 units/mL penicillin, and 100 μg/mL streptomycin. MCF10A cells were cultured in a mammary epithelial cell medium (MEpiCM, ScienCell). CAFs were maintained in DMEM/F12 (Hyclone, USA) containing 10% FBS, 100 units/mL penicillin and 100 μg/mL streptomycin. All the cell lines were cultured in a humidified atmosphere of 5% CO_2_ at 37 °C. For hypoxia, the cells were incubated in a chamber with 1% O_2_.

### Breast cancer samples

Fresh breast cancer tissues and their adjacent non-tumor tissues were acquired from Zhongshan Hospital, Fudan University (Shanghai, China). The studies involving human participants were reviewed and approved by the ethics committee. The patients provided written informed consent to participate in this study. All the tissues were stored at −80 °C for use.

### Tumor stroma CAFs isolation

The fresh tissues from the breast cancer patients were collected in 10 ml phosphate-buffered saline (PBS) supplemented with 2% (double-strength) penicillin–streptomycin and 0.1% (0.25 μg/mL) amphotericin B (Fungizone; Thermo Fisher Scientific, Waltham, USA) and washed 3 times with PBS. Tissues were cut into 2–3 mm^3^ fragments in sterile conditions and then cultured in 6-well plates containing DMEM/F12 (Hyclone, USA) supplemented with 20% FBS, 2% penicillin–streptomycin and 0.1% amphotericin B. The cells were cultured in a chamber with 5% CO_2_ at 37 °C. The growth medium was changed every 3–4 days. Fibroblasts were regularly expanded in the standard manner.

### Exosome purification

Exosomes were harvested from cell culture supernatants, tissue, and blood and RNA isolated. Exosomes were prepared essentially as described. Briefly, CAFs or NFs were cultured with media without FBS under hypoxia (1% O_2_) or normoxia (21% O_2_). After 48 h, supernatant fractions were collected from the cells. The supernatant was centrifugated at 3000 rpm for 20 min, the exosomes were isolated by Total Exosome Isolation Reagent (Invitrogen, USA). The exosomes for circRNA array by ultracentrifuge. The exosomes were stored at −80 °C for further experiments.

### Transmission electron microscopy

Exosomes from CAFs were extracted and re-suspended with low salt PBS and dropped onto a 100-mesh copper grid. The samples were negatively stained by 2% phosphotungstic acid for 3 min, allowed to dry for 15–20 min, and examined under a transmission electron microscope (TEM) (FEI Tecnai G2 Spirit, Thermo Fisher Scientific) at 80 kV. The photos of exosomes were taken under the TEM.

### Luciferase reporter assay

The wild-type (WT) and mutant fragments in 3′-UTR of circHIF1A related to the miRNA-580-5p binding site were designed, synthesized, and inserted into pGL4-basic vectors. Cells were seeded in a 96-well plate, and the cells were co-transfected with pGL4-basic vectors (50 ng), Renila luciferase reporter vectors (5 ng) (pRL-TK), and miRNA-580-5p mimics or inhibitor or circHIF1A overexpressing lentivirus. After 48 h, the luciferase activity was measured with a dual-luciferase reporter assay system (Promega). The luciferase values were normalized to the corresponding Renila luciferase values, and then the fold changes were calculated.

### Biotin-coupled miRNA capture

The 3′ end biotinylated miR-580-5p mimics or control RNA (Ribio, Guangzhou, China) were transfected into cells (2 × 10^6^/well) at a final concentration of 20 nM for 48 h before harvest.

### circHIF1A overexpression, shRNAs, miRNA inhibitors, and siRNAs

To generate a circHIF1A-overexpressing cell line, we stably transfected CAFs, with the pLCDH-circHIF1A plasmid (Genechem, Shanghai, China). To generate circHIF1A-downregulation cells, we transfected CAFs cells, which express high levels of circHIF1A, with circHIF1A shRNA (Genechem, Shanghai, China) targeting the ring-forming sequences to break up its circular structure. After transfection, the circHIF1A overexpression and silenced cell lines were selected by culture in the presence of 2 μg/mL puromycin. miR-580-5p inhibitor or mimics were ordered from Genechem (Shanghai, China). CD44 overexpression lentivies vectors were also ordered from Genechem (Shanghai, China).

### Sphere formation assay

SKBR3 and MDA-MB-231 cells (5000 cells/well) were cultured in a 6-well ultra-low attachment plate (Corning Inc., USA). These cells were cultivated for 7–10 days in a stem cell medium with 20 ng/ml EGF and 20 ng/ml bFGF and 1× B27 (Thermo Scientific, USA, IL) at 37 °C under 5% CO_2_. The mammospheres with a diameter larger than 50 μm were counted under inverted microscopy or harvested by centrifugation for other experiments.

### Western blot analysis

Cells were lysed in a lysis buffer and centrifugated by 4000*g* for 30 min at 4 °C. BCA method was used to detect the protein concentration. Ten microgram protein lysate of exosomes or 50–100 µg protein lysate was separated by sodium dodecyl sulphate-polyacrylamide gel electrophoresis and transferred onto polyvinylidene fluoride (PVDF) (GE life). The PVDF with protein was probed with primary antibodies CD81, CD63, HSP70, CD44, SOX2, ALDH1, OCT4, Nanog, HIF-1α, GAPDH, and the secondary antibodies (CST, USA). Then, the PVDF was stained with horseradish peroxidase-labeled secondary antibodies. The protein was visualized using an enhanced chemiluminescence detection kit (Absin, China).

### Immunofluorescent staining of cells

Cells were grown on sterile glass coverslips overnight in a 37 °C culture incubator. Prior to immunofluorescent staining, the cells were fixed in pre-chilled −20 °C methanol for 5 min and then incubated with 5% normal serum in PBS at 37 °C for 30 min to block nonspecific binding of IgG. The cells were then incubated with the desired primary antibodies in PBS with 1% normal serum at 4 °C overnight. After washing the cells twice with PBS, fluorescence-conjugated secondary antibody and 4′,6-diamidino-2-phenylindole (DAPI, Roche, USA) were added onto the coverslips, for 1.5 h. Fluorescently stained cells were examined under a fluorescence microscope.

### CircRNA array and data analysis

Arraystar Human circRNA Array V2 analysis of six samples was performed. Total RNA from each sample was quantified using the NanoDrop ND-1000. All the samples were performed for quality control. The sample preparation and microarray hybridization were performed based on Arraystar’s standard protocols. Briefly, total RNAs were digested with Rnase R (Epicentre, Inc.) to remove linear RNAs. The enriched circRNAs were amplified and transcribed into fluorescent cRNA using Arraystar Super RNA Labeling Kit (Arraystar). The labeled cRNAs were hybridized onto the Arraystar Human circRNA Array V2 (8x15K, Arraystar). The slides were washed and the arrays were scanned by the Agilent Scanner G2505C. Hierarchical Clustering was used to show the distinguishable circRNAs expression.

### RNA FISH

Cy3-labeled oligonucleotide probe for circHIF1A and FAM-labeled oligonucleotide probe for miR-580-5p were applied for RNA FISH. The oligonucleotide sequences are available in the Supplementary file Table 1. The paraffin section of breast cancer samples was deparaffinized with 100% xylene and rehydrated with different graded ethanol. For RNA FISH of co-localization of circHIF1A and miR-580-5p, cells were seeded in a glass-bottom dish. Then they were incubated with prehybridization solution at 37 °C for 30 min and the probes (Ribobio, China, 20 μM) were added to slides or dishes individually and hybridized overnight. Then they were washed with buffer I (4 × saline sodium citrate (SSC), 0.1% Tween-20) three times, wash with buffer II (2 × SSC) for once, and wash with buffer III (1 × SSC) for once. After being washed with PBS, they were incubated with DAPI to stain the cell nuclear.

### RNA-binding protein immunoprecipitation (RIP)

The RIP assay was performed using EZ-Magna RIP Kit (Millipore, USA). Briefly, MDA-MB-231 (1 × 10^7^) cells were incubated with lysis buffer with protease and RNase inhibitors. Then the cell lysis was incubated with magnetic beads which are conjugated with human ani-AGO2 antibody (Millipore, USA) or negative control IgG (Millipore, USA) at 4 °C overnight, washed, incubated with Proteinase K, and finally, the immunoprecipitated RNA was purified for real-time PCR analysis.

### CircRNA pull-down

CircRNA pull-down assay was performed using PIERCE MAGNETIC RNA Pull-down Kit (Thermo, USA). Biotin-labeled circHIF1A probe and its control probe were synthesized (Sangon Biotech, China). Cells were cross-linked by 1% formaldehyde for 30 min, lysed in co-IP buffer, and centrifugated. The supernatant was incubated with circHIF1A-specific probes-streptavidin beads (Life Technologies, USA) mixture overnight at 37 °C. The next day, the samples were washed and incubated with lysis buffer and proteinase K. RNA was extracted from the mixture. CircHIF1A, miR-580-5p, and β-actin were analyzed.

### Ribonuclease R (RNase R) treatment

Total RNA was incubated with or without 3 U/mg RNase R (Epicentre, San Diego, CA, USA) at 37 °C according to the manufacturer’s instructions. The resulting RNA was purified with the RNeasy MinElute Cleanup Kit (Qiagen) for further analysis.

### Real-time RT-PCR

Total RNA was extracted from the cells with the indicated treatment using EZ total RNA isolation kit according to the manufacturer’s protocol. RNA was qualified and performed for real-time reverse transcription-polymerase chain reaction (RT-PCR) analysis. The primer sequences using in this study were provided in the supplementary material (Additional File 1, Table S1). The PCR was run on the Roche 480 Real-Time PCR System using the following thermocycling parameters: 95 °C for 10 min, 40 cycles at 95 °C for 10 s, 60 °C for 30 s, 72 °C for 10 s, followed by a melting curve analysis. The relative circRNA, miRNA, or mRNA levels were calculated by comparing Ct values of the samples with those of the reference, all data normalized to β-actin or U6 snRNA.

### Cell proliferation assay

Breast cells were treated with exosomes and then seeded in a 96-well plate. Cell proliferation was evaluated by CCK8 assay. The survival rates were analyzed. For colony formation, the cells were seeded in 6-well plates for 10–14 days and the colonies with more than 50 cells were counted and analyzed.

### Immunofluorescent staining assay

Cells were grown in 24-well plates, fixed in pre-chilled −20 °C methanol for 5 min, permeabilized in 0.2% Triton X 100, and then incubated with 5% BCA in PBS at 37 °C for 30 min to block nonspecific binding of IgG. The cells were then incubated with the desired primary antibodies including α-SMA in PBS with 1% BCA at 4 °C overnight, and then the cells were stained with fluorescence-conjugated secondary antibody and 4′,6-diamidino-2-phenylindole (DAPI, Roche, USA). Cells were examined under a fluorescence microscope.

### Xenograft model

Six-week-old female BALB/c nude mice were housed under the standard conditions. CAFs were infected with lentivirus circHIF1A shRNA or the control and selected in the presence of puromycin (1 μg/ml) and the exosomes were collected for injecting into the models. MDA-MB-231 cells (1 × 10^7^) suspended in 100 μl Matrigel with 200 μg exosomes purified from the culture supernatants of exosomes from NFs by subcutaneously injecting into the fat pad (6 mice per group). Tumor volume was monitored twice a week and calculated using the formula: volume (mm^3^) = (width^2^ × length)/2. After 4 weeks following the inoculation, the mice were sacrificed and tumor samples were weighted and harvested for histological analysis. All animal experiments were performed under approval by the Shanghai Medical Experimental Animal Care Commission.

### Statistical analysis

Statistical analyses were performed using SPSS 16.0 and Excel. All quantitative experiments were repeated with at least three independent biological repeats and are presented as the means ± SD (standard deviation). Quantitative data were analyzed by either one-way analysis of variance (ANOVA) (multiple groups or parametric generalized linear model with random effects for tumor growth and CCK8 assay) or *t*-test (two groups). *p* < 0.05 was considered statistically significant.

## Results

### The circRNAs profile of the exosomes from CAFs in hypoxic stress

**Fig. 1:**
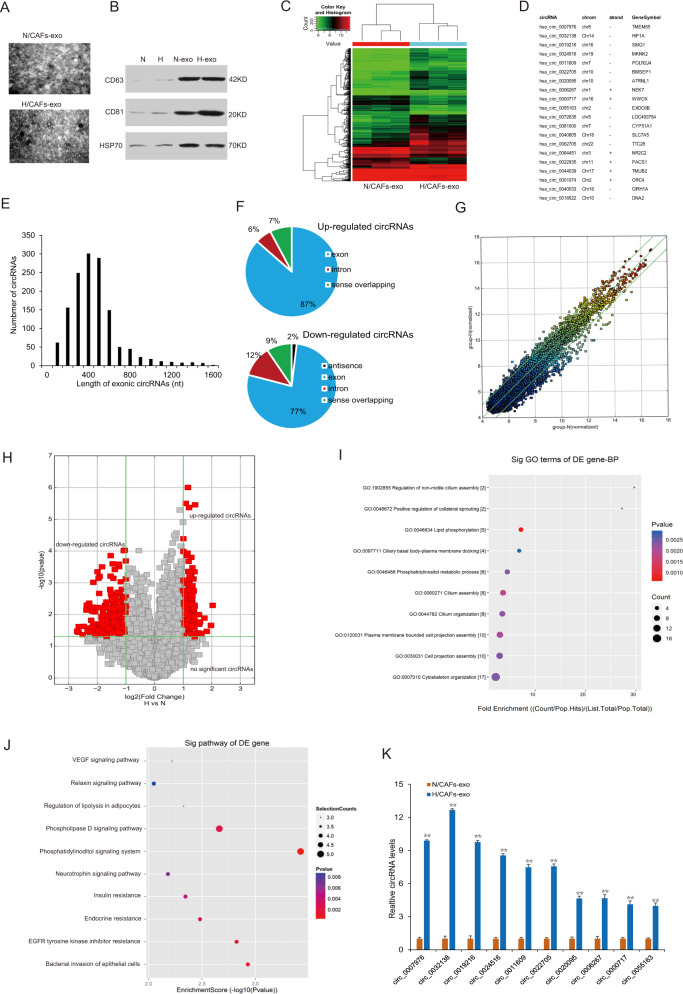
The circRNAs profile of the exosomes from CAFs in hypoxia stress. **A** The representative pictures of exosomes from CAFs in hypoxia and normoxia. **B** The exosome markers were identified by western blotting. **C** The heat map showed the most significant circRNAs in exosomes from CAFs in the presence of hypoxia and normoxia. CircRNA expression profiles were performed by circRNA array. The map showed the differentially expressed circRNAs over two-folds change. The red color indicated high expression level, and the green color indicated a low expression level. **D** The top ten different circRNAs in the hypoxic exosomes from normoxic exosomes. **E** circRNA distribution in hypoxia CAFs and normoxia CAFs. **F** The resources of circRNAs. **G** The scatter plot was used to assess the variation in circRNA expression between H-exo and N-exo. The values of *x-* and *y*-axes in the scatter plot were the normalized signal values of the samples (log2 scaled). The green lines were fold-change lines. **H** Volcano plot showed the differential circRNAs in exosomes of hypoxic and normoxic CAFs. Left red indicated the down-regulated circRNAs. Right red indicated the upregulated circRNAs. **I** KEGG analysis of circRNAs in H-exo vs. N-exo. **J** GO analysis of circRNAs in hypoxia H-exo vs. N-exo. **K** The top ten upregulated circRNAs in the exosomes from hypoxic CAFs. *p < 0.05, **p < 0.01.

To investigate the effect of CAFs exosomal circular RNAs on the breast cancer cellular phenotypes, CAFs from breast cancer tissues were cultured in hypoxia (H-exo) and normoxia (N-exo) for 48 h, and the exosomes were taken photos under a TEM (Fig. 1A). The exosome markers were identified (Fig. 1B). To find different circRNAs in exosomes between hypoxic CAFs and normoxic CAFs, circRNA array was done and the cluster heat map was shown (Fig. 1C). The top ten significant upregulated and downregulated circRNAs in hypoxic exosomes from CAFs were listed compared to the normoxic CAFs (Fig. 1D). CircRNAs was detectable and the length of most circRNAs was less than 1000 nucleotides in both H-exo and N-exo (Fig. 1E). Most of circRNAs in the exosomes of CAFs were from exons (Fig. 1F). The scatter plot and volcano plot was used to assess the variation in circRNA expression between H-exo and N-exo from CAFs (Fig. 1G, H). Disease pathway analysis suggested that these differentially expressed circRNAs were relevant to several vital physiological processes, cellular components, molecular functions, and critical signaling pathways (Fig. 1I, J). The expression of the top ten upregulated circRNAs including has_circ_0007976 (TMEM65), has_circ_0032138 (HIF1A), has_circ_0019216 (SMG1), has_circ_0024516 (MKNK2), has_circ_0011609 (POLR2J4), has_circ_0022705 (BMSEP1), has_circ_0020095 (ATRNL1) has_circ_0006267 (NEK7), has_circ_0000717 (WWOX), and has_circ_0055163 (EXOC6B) in the exosomes from hypoxic CAFs were verified and the result indicated that has_circ_0032138 (circHIF1A) was significantly upregulated in exosomes from hypoxic CAFs. It was also verified that exosomal circHIF1A was upregulated in hypoxia CAFs than in normoxic CAFs (Fig. 1K), so circHIF1A was selected for the next investigation.

### The characteristics of circHIF1A in breast cancer

**Fig. 2:**
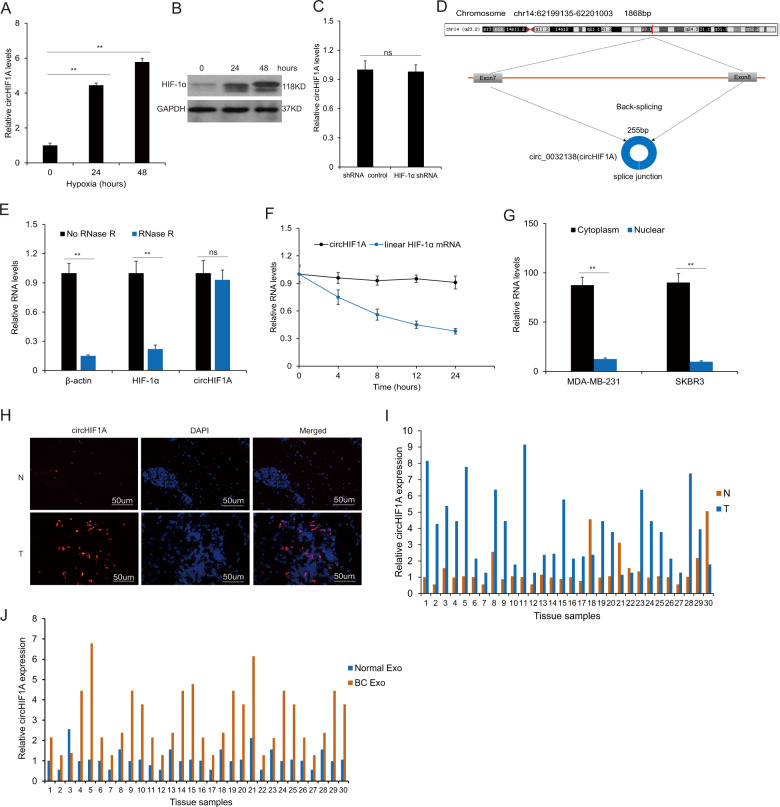
The characteristics of circHIF1A abundance in breast cancer. **A** CircHIF1A levels increased in hypoxic CAFs. CAFs were grown in hypoxia (1% O_2_) and the exosomes were extracted for circHIF1A expression examination. **B** HIF-1α expression in hypoxic exosomes from CAFs. CAFs were grown in hypoxia (1% O_2_) and total protein was extracted for HIF-1α by western blotting. **C** circHIF1A expression in CAFs with HIF-1α downregulation. **D** The genomic loci of the HIF-1α gene and circHIF1A. **E** qRT-PCR analysis of circHIF1A and HIF-1α RNA after treatment with RNase R in CAFs. **F** qRT-PCR analysis of circHIF1A and HIF-1α RNA after treatment with Actinomycin D at the indicated time points in CAFs. **G** qRT-PCR analysis of circHIF1A in the cytoplasm or the nucleus in MDA-MB-231 and SKBR3 cells. **H** The circHIF1A expression levels were increased in breast cancer tissues compared with the normal tissues. CircHIF1A expression was evaluated by FISH. The nuclei were stained with DAPI for the blue color, and the cytoplasmic circHIF1A was stained with red color. **I** CircHIF1A expression in the breast cancer tissues. CircHIF1A expression was based on the score in breast cancer tissues. **J** CircHIF1A expression in the blood exosomes from breast cancer patients. Normal Exo exosomes from blood of healthy donors, BC exo exosomes from blood of the patients with breast cancer. *p < 0.05, **p < 0.01.

To learn the feature of circHIF1A in breast cancer stroma cells, the hypoxia exosomes from CAFs under hypoxia (1% O_2_) were used for RNA extraction for analyzing circHIF1A. The real-time RT-PCR data showed that circHIF1A expression increased in time-dependent under hypoxia (Fig. 2A). HIF-1α protein levels were evaluated in hypoxic CAFs (Fig. 2B), however, circHIF1A was not influenced in CAFs with HIF-1α inhibition (Fig. 2C). The spliced circHIF1A length was 255 bp, which located on chr14:62199135-62201003. The genomic loci of the HIF-1α gene and circHIF1A were shown (Fig. 2D). To investigate whether circHIF1A responded to RNase R, qRT-PCR analysis of circHIF1A and HIF-1α RNA after treatment with RNase R in breast cancer cells and it was shown that circHIF1A was resistant to RNase R (Fig. 2E). To know the RNA stability of circHIF1A and HIF-1α mRNA, the cells were treated with Actinomycin D at the indicated time points in breast cancer cells and the data showed that the half survival time of circHIF1A was not influenced (Fig. 2F). It was also shown that circHIF1A was mainly present in the cytoplasm of cells (Fig. 2G). CircHIF1A expression was identified in breast cancer tissues. The expression of circHIF1A was upregulated in the breast cancer tissues by FISH (Fig. 2H). CircHIF1A was upregulated in the breast cancer tissues (*n* = 30) compared to the adjacent normal tissues by RT-PCR analysis (Fig. 2I). There was a consistent result in the exosomes from the blood of patients (*n* = 30) (Fig. 2J).

### Hypoxic stroma exosomes circHIF1A promoted breast cancer cell proliferation and stemness

**Fig. 3:**
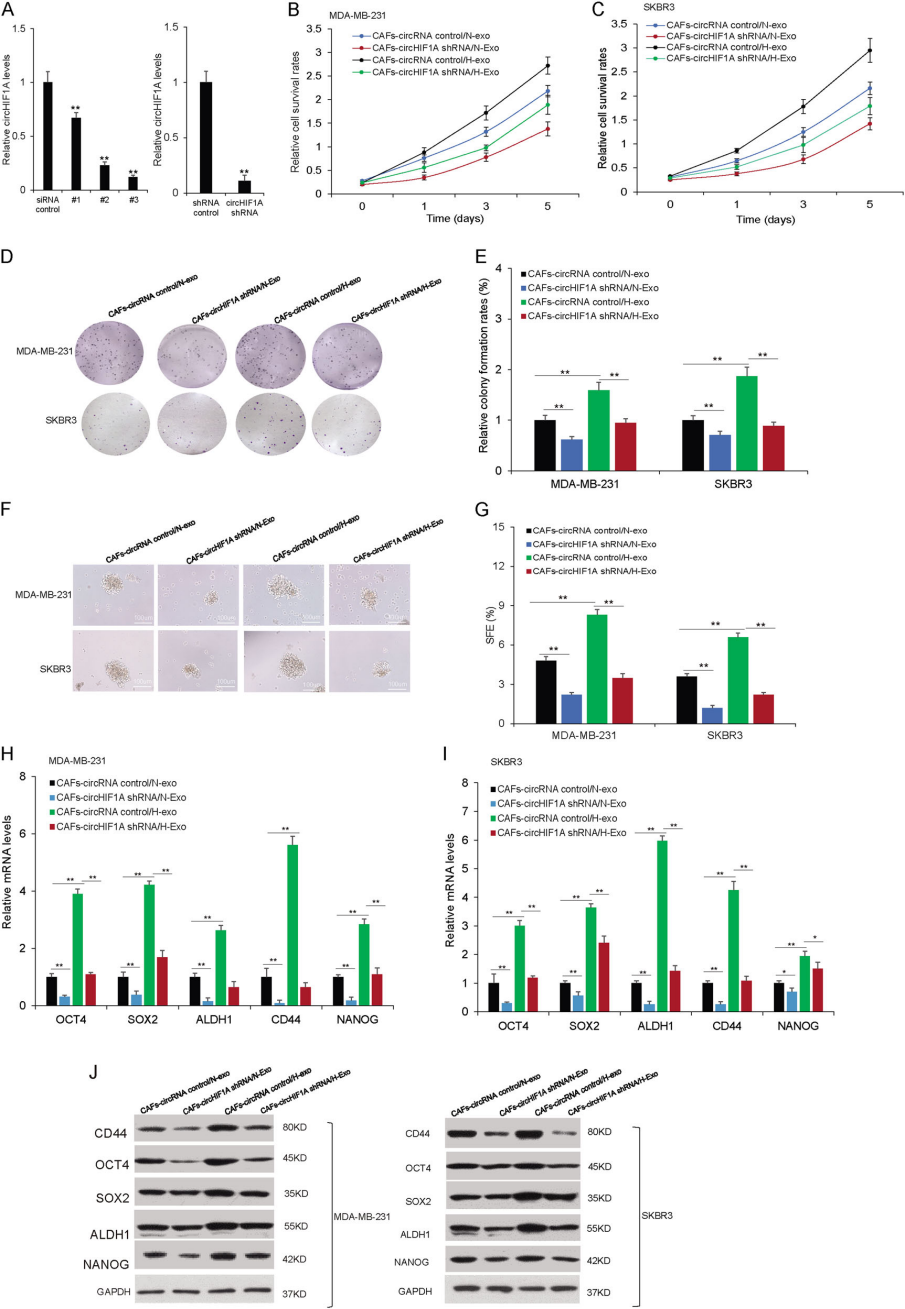
Hypoxic stroma exosomes circHIF1A promoted breast cancer cell stemness. **A** CircHIF1A was also lower in the exosomes. Cells with circHIF1A down-regulation and their exosomes were isolated and the RNA for circHIF1A measurement by real-time RT-PCR. **B**, **C** Cell proliferation in MDA-MB-231 and SKBR3 cells with exosomes from CAFs with CAFs-circRNA control/N-Exo, CAFs-circRNA control/H-Exo, CAFs-circHIF1A shRNA/N-Exo, or CAFs-circHIF1A shRNA/H-Exo treatments. The survival ability was suppressed by the CCK8 assay. **D** Cell proliferation in MDA-MB-231 and SKBR3 cells with exosomes from CAFs-circRNA control/N-Exo, CAFs-circRNA control/H-Exo, CAFs-circHIF1A shRNA/N-Exo, or CAFs-circHIF1A shRNA/H-Exo treatments. The survival ability was assayed by colony formation. **E** The data analysis from (**D**). **F** Mammosphere formation in breast cancer cells. MDA-MB-231 and SKBR3 cells were exposed to exosomes from CAFs in hypoxia or normoxia conditions. Sphere numbers became more in H-exo, and downregulation of circHIF1A reduced the numbers. The photos of mammospheres were taken under a microscope. **G** The data were analyzed from (**F**). **H**, **I** The stem cell markers decreased in the presence of H-exo with circHIF1A downregulation in MDA-MB-231 and SKBR3 cells. MDA-MB-231 and SKBR3 cells were in the presence of exosomes from CAFs-circRNA control/N-Exo, CAFs-circRNA control/H-Exo, CAFs-circHIF1A shRNA/N-Exo, or CAFs-circHIF1A shRNA/H-Exo for 6 days, and then total RNA was extracted from real-time RT-PCR. **J** CircHIF1A regulated stem cell-associated gene expression on protein levels. MDA-MB-231 cells and western blotting. MDA-MB-231 and SKBR3 cells were in the presence of exosomes from CAFs (CAFs-circRNA control/N-Exo, CAFs-circRNA control/H-Exo, CAFs-circHIF1A shRNA/N-Exo, or CAFs-circHIF1A shRNA/H-Exo) for 6 days, and then total protein was extracted for western blot. *p < 0.05, **p < 0.01.

To know the role of circHIF1A in breast cancer cellular functions, it was knocked down by siRNAs (different siRNA target sequence #1, #2, and #3) and circHIF1A level was lower in the exosomes from CAFs (Fig. 3A). circHIF1A siRNAs (#3) were chosen for circHIF1A shRNA with the lentivirus system. CAFs with circHIF1A downregulation and their control were cultured under hypoxia or normoxia, and the exosomes were prepared (CAFs-circRNA control/N-Exo, CAFs-circRNA control/H-Exo, CAFs-circHIF1A shRNA/N-Exo, or CAFs-circHIF1A shRNA/H-Exo). Breast cancer cells were co-cultured with the above exosomes, and cell survival ability was assayed by CCK8 and it was found that cell survival ability was increased in CAFs-circRNA control/H-Exo compared with cells in CAFs-circRNA control/H-Exo and suppressed in the cells with CAFs-circHIF1A shRNA/H-Exo (Fig. 3B, C). The colony formation assay also verified the results (Fig. 3D, E). To know the role of circHIF1A in breast cancer stem cell plasticity, MDA-MB-231, and SKBR3 cells were exposed to exosomes from CAFs in hypoxia or normoxia conditions. Mammospheres became more in H-CAFs/exo, and downregulation of circHIF1A reduced the mammosphere numbers (Fig. 3F, G). The stem cell markers OCT4, SOX2, ALDHA1, CD44, and Nanog decreased in the presence of H-CAFs-exo with circHIF1A low levels by real-time RT-PCR (Fig. 3H, I) and western blotting (Fig. 3J).

### CircHIF1A served as a sponge for miR-580-5p in breast cancer cells

**Fig. 4:**
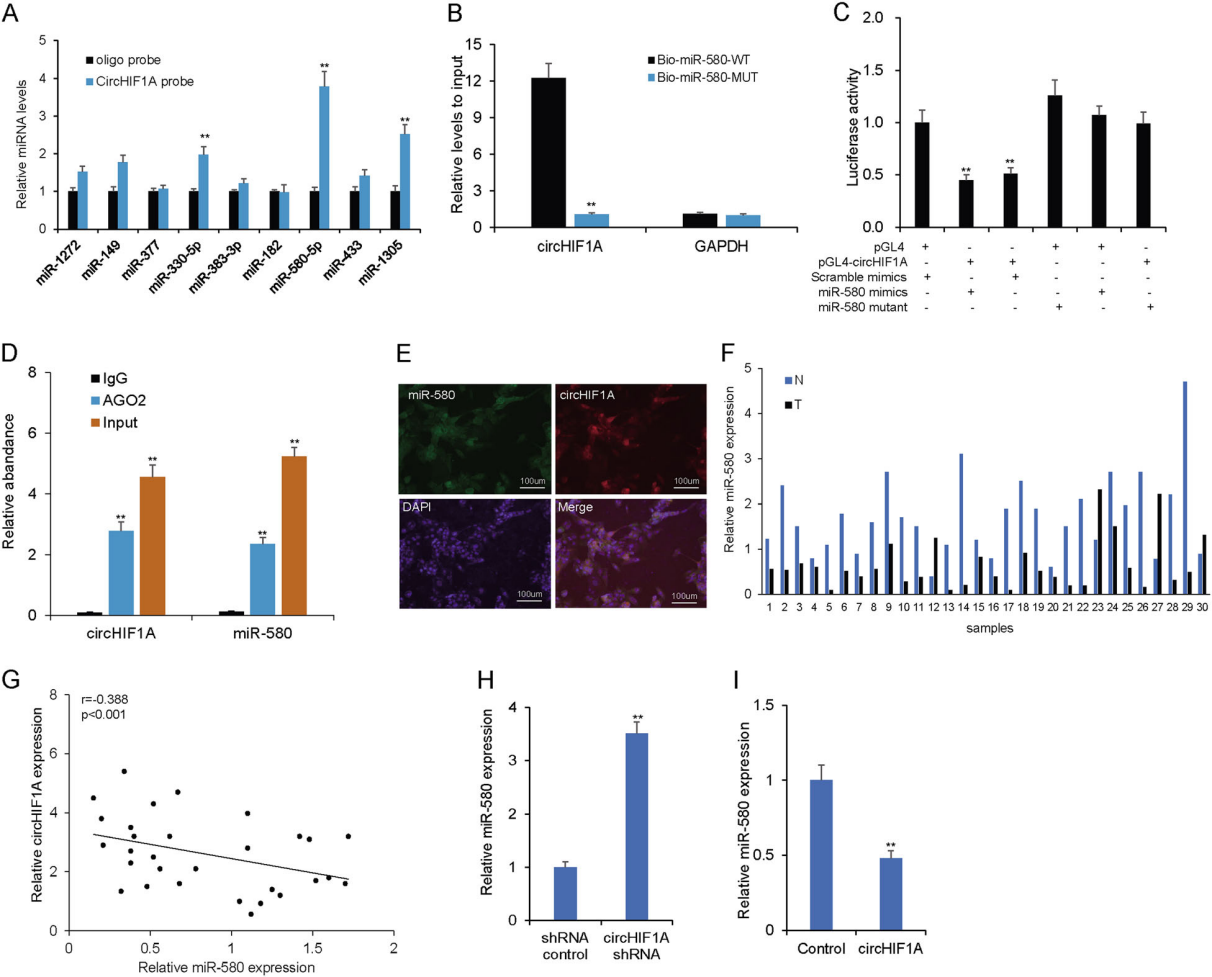
CircHIF1A served as a sponge for miR-580-5p in breast cancer cells. **A** Quantitative real-time PCR analysis of circHIF1A expression in lysates of SKBR3 cells with circHIF1A overexpression following biotinylated-circHIF1A pull-down assay. **B** Biotinylated WT/mutant miR-580 was transfected into SKBR3 cells with circHIF1A overexpression. After streptavidin capture, circHIF1A expression was detected by quantitative real-time PCR. **C** Luciferase activity in SKBR3 cells co-transfected with luciferase reporters containing circHIF1A sequences with WT or mutated miR-580 binding sites and mimics of miR-580 or controls. **D** RNA immunoprecipitation (RIP) assay demonstrated that AGO2 could the enrichment of circHIF1A and miR-580 in MDA-MB-231 cells compared to anti-IgG. **E** Co-localization of circHIF1A with miR-580 in MDA-MB-231 cells. **F** Quantitative real-time PCR analysis of miR-580 expression in breast cancer tissues. **G** Negative relationship between circHIF1A and miR-580 in cancer tissues. **H**, **I** MiR-580 expression in MDA-MB-231 cells. MDA-MB-231 cells were transfected with circHIF1A siRNA or circHIF1A overexpression, and then total RNA was extracted for real-time RT-PCR of miR-580. *p < 0.05, **p < 0.01.

CircHIF1A locates mainly in the cytoplasm, so the molecular mechanism may be as a miRNA sponge. To study the ceRNA mechanism of circHIF1A, the interaction of circHIF1A and miRNA were predicted, and the potential miRNAs including miR-1272, miR-149, miR-377, miR-330-5p, miR-383-3p, miR-182, miR-580-5p, miR-433, and miR-1305 based on circular RNA Interactome (https://circinteractome.nia.nih.gov/index.html) (Supplementary Fig. S1). To investigate these potential target miRNAs, we designed a 3′-terminal-biotinylated-circHIF1A probe that was verified to pull down circHIF1A in SKBR3 cells, and overexpression of circHIF1A enhanced the pulldown efficiency (Fig. 4A). Quantitative real-time PCR analysis revealed that miR-580-5p (named as miR-580 in the following study) was abundantly pulled down by the circHIF1A probe in SKBR3 cells (Fig. 4B). In luciferase reporter assays, we demonstrated that miR-580 overexpression dramatically reduced the luciferase activity of the cells transfected with the vector containing the complete circHIF1A sequence, but did not influence the luciferase activity of cells transfected with the vector containing the mutant miR-580 binding site (Fig. 4C). The bioinformatics predictions showed that a high degree of AGO2 occupancy in the region of circHIF1A (Supplementary Fig. S2). RIP assay indicated that AGO2 bond to circHIF1A and miR-580 by anti-AGO2 antibody, not by IgG (Fig. 4D). FISH studies showed that circHIF1A and miR-580 were co-localized in the cytoplasm in breast cancer cells (Fig. 4E). Quantitative real-time PCR also showed that miR-580-5p was significantly downregulated in tissues (Fig. 4F). There was a negative relationship between circHIF1A and miR-580 in cancer tissues (Fig. 4G). When the cells were overexpressed circHIF1A or downregulation of circHIF1A, miR-580 deceased or upregulated in breast cancer cells (Fig. 4H, I). The data suggested that circHIF1A was a miR-580 sponge in breast cancer cells.

### CircHIF1A regulated CD44 via miR-580-5p in breast cancer cells

**Fig. 5:**
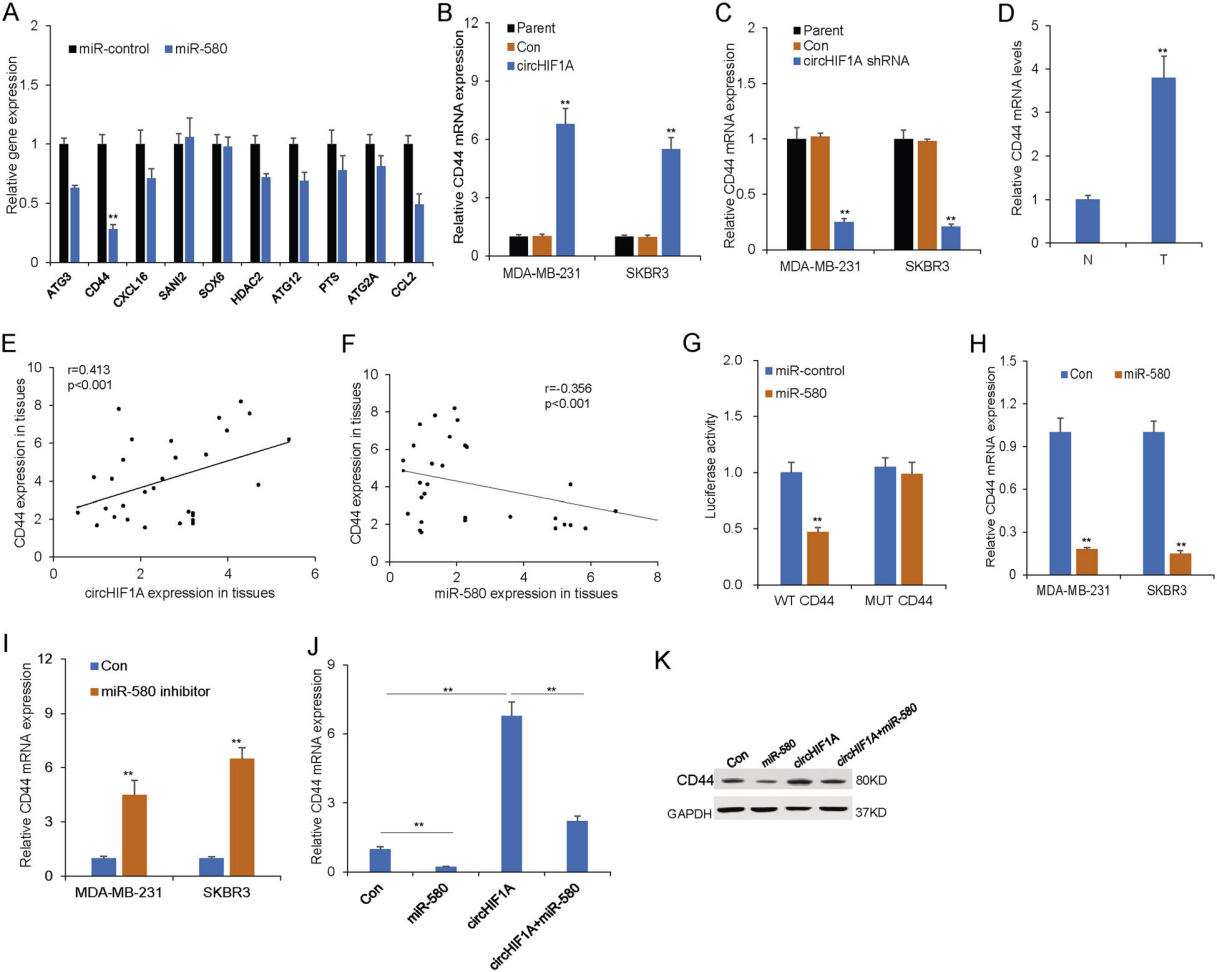
miR-580-5p regulated CD44 expression in breast cancer by circHIF1A modulation. **A** The expression of predicated 10 genes in MDA-MB-231 cells. **B** CD44 expression levels were increased by ectopic expression of circHIF1A in MDA-MB-231 cells. Parent: MDA-MB-231 cells; Con: circHIF1A control. **C** CD44 expression level decreased by inhibiting circHIF1A expression in MDA-MB-231 cells. Parent: MDA-MB-231 cells; Con: circHIF1A control. **D** CD44 was upregulated in breast cancer tissues compared with normal adjacent tissues. N normal adjacent tissues, T breast cancer tissues. **E** A positive correlation of the expression of circHIF1A and CD44 in breast cancer specimens. **F** A negative correlation was observed between miR-580 levels and CD44 levels in breast cancer specimens. **G** Co-transfection of MDA-MB-231 cells with miR-580 mimics and reporter plasmids significantly reduced the luciferase activity of CD44. **H** Expression of CD44 was significantly upregulated following treating with a miR-580 inhibitor. **I** Expression of CD44 was significantly down-regulated in MDA-MB-231 cells with miR-580 overexpression. **J** CD44 expression in MDA-MB-231 cells with circHIF1A or miR-580 expression by real-time RT-PCR. **K** CD44 expression in MDA-MB-231 cells with circHIF1A or miR-580 expression by western blotting. *p < 0.05, **p < 0.01.

There is accumulating evidence that miR-580-5p simultaneously targets numerous oncogenes. Therefore, we investigated the ability of circHIF1A to exert a pro-tumor role by modulating the expression of miR-580 targeting oncogenes. Of these, we selected ten genes that exhibited significantly dysregulated expression in MDA-MB-231 cells (Fig. 5A). Expression of CD44 increased by ectopic expression of circHIF1A (Fig. 5B) but decreased by inhibiting circHIF1A expression (Fig. 5C). It was confirmed that CD44 was upregulated in breast cancer tissues compared with normal adjacent tissues by qRT-PCR (Fig. 5D). Pearson correlation analysis showed a positive correlation of the expression of circHIF1A and CD44 in breast cancer specimens (Fig. 5E). A negative correlation was observed between the expression of miR-580 and CD44 in breast cancer specimens (Fig. 5F). To confirm that miR-580-5p regulated CD44, we constructed a luciferase-based reporter containing WT and mutated 3′-UTR sequences of CD44. Co-transfection of cells with miR-580 mimics and reporter plasmids significantly reduced the luciferase activity of CD44. However, co-transfection of miR-580 mimics and mutated vectors had no evident effect on the luciferase activity in MDA-MB-231 cells (Fig. 5G). These results imply that miR-580 degrades CD44 mRNA by targeting its 3′-UTR and that this degradation can be blocked by the sponge activity of circHIF1A. In MDA-MB-231 cells with miR-580 inhibition, transcription of CD44 was increased (Fig. 5H). In contrast, CD44 was significantly up-regulated following miR-580 overexpression on the transcriptional level (Fig. 5I). In contrast, CD44 was significantly up-regulated following circHIF1A overexpression and decreased with miR-580 overexpression on the transcriptional level (Fig. 5J). Western blot analysis indicated that CD44 expression was regulated by circHIF1A or miR-580 in MDA-MB-231 cells (Fig. 5K). The data indicated that CD44 was a target gene of miR-580 and was regulated by circHIF1A.

### CircHIF1A regulated cancer cell stemness by CD44

**Fig. 6:**
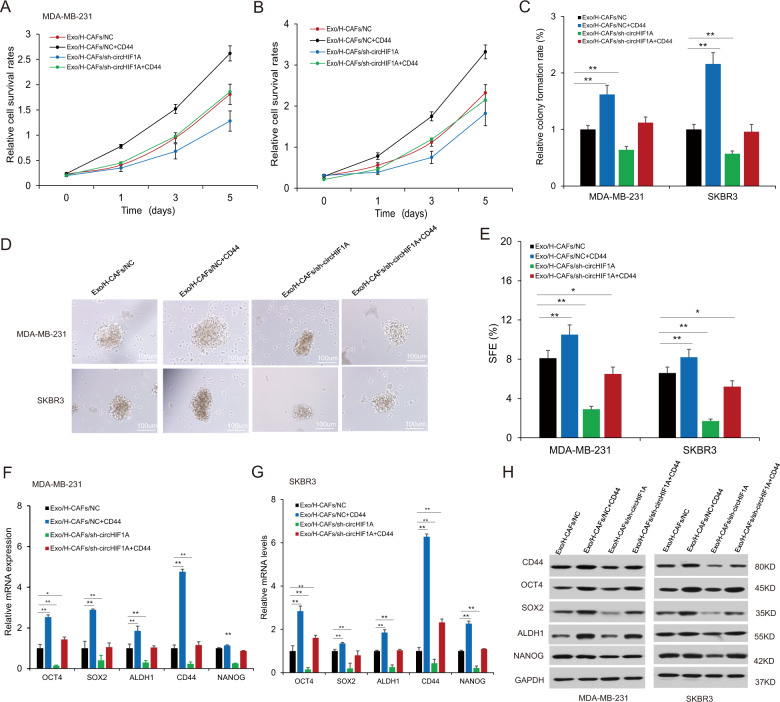
CircHIF1A regulated cancer cell stemness by CD44. **A**, **B** Cell proliferation in breast cancer cells in the presence of hypoxia exosomes from CAFs. MDA-MB-231 and SKBR3 cells were overexpressed CD44 and treated with the exosomes (Exo/H-CAFs/NC, Exo/H-CAFs/sh-circHIF1A, Exo/H-CAFs/NC + CD44, and Exo/H-CAFs/sh-circHIF1A + CD44). The survival ability was assayed by CCK8. **C** Cell proliferation in breast cancer cells in the presence of hypoxia exosomes from CAFs. MDA-MB-231 and SKBR3 cells were overexpressed CD44 and treated with the exosomes (Exo/H-CAFs/NC, Exo/H-CAFs/sh-circHIF1A, Exo/H-CAFs/NC + CD44, and Exo/H-CAFs/sh-circHIF1A + CD44). The survival ability was assayed by colony formation. **D** Mammosphere formation in breast cancer cells. MDA-MB-231 and SKBR3 cells were overexpressed CD44 and treated with the exosomes (Exo/H-CAFs/NC, Exo/H-CAFs/sh-circHIF1A, Exo/H-CAFs/NC + CD44, and Exo/H-CAFs/sh-circHIF1A + CD44). The photos of mammospheres were taken under a microscope. **E** The data were analyzed from (**D**). **F**, **G** The stem cell markers CD44, ALDHA1, OCT4, CD44, and Nanog in breast cancer cells in the presence of H-CAFs-exo (Exo/H-CAFs/NC, Exo/H-CAFs/sh-circHIF1A, Exo/H-CAFs/NC + CD44, and Exo/H-CAFs/sh-circHIF1A + CD44). The expression was assayed by real-time RT-PCR. **H** The stem cell markers CD44, ALDHA1, OCT4, SOX2, and Nanog in breast cancer cells in the presence of exosomes derived CAFs (Exo/H-CAFs/NC, Exo/H-CAFs/sh-circHIF1A, Exo/H-CAFs/NC + CD44, and Exo/H-CAFs/sh-circHIF1A + CD44). The expression was assayed by western blotting. NC negative control. *p < 0.05, **p < 0.01.

To know the role of circHIF1A in breast cancer cell plasticity by CD44, breast cancer cells were in the presence of hypoxic CAFs exosomes with down-regulation of circHIF1A or CD44 overexpression in breast cancer cells, and cell survival ability was assayed by CCK8 and it was found that cell survival ability was suppressed in the MDA-MB-231 and SKBR3 cells with circHIF1A shRNA exosomes derived CAFs and CD44 could rescue the progression partially (Fig. 6A, B). Using colony formation assay, it was shown cell colonies were reduced in the MDA-MB-231 and SKBR3 cells with circHIF1A shRNA exosomes derived CAFs and CD44 could rescue the reduction partially (Fig. 6C). Mammosphere formation was assayed for assessing stem cell properties. Mammospheres became less in the cells treated with CAFs exosomes with downregulation of circHIF1A and introduction of CD44 into the breast cancer cells could rescue the reduced sphere numbers (Fig. 6D, E). To evaluate the gene expression of stem cells associated molecules, MDA-MB-231 and SKBR3 cells were overexpressed CD44 and then treated with the exosomes from CAFs with or without circHIF1A downregulation, the stem cell markers including OCT4, SOX2, ALDHA1, CD44, and Nanog in the presence of H-CAFs-exo with circHIF1A downregulation decreased and CD44 overexpression could rescue the decrease partially in breast cancer cells on transcriptional levels (Fig. 6F, G) and post-transcriptional levels (Fig. 6H). These data suggested that circHIF1A from CAFs exosomes in hypoxia enhanced breast cancer stem cell plasticity in the tumor microenvironment by upregulation of CD44.

### Biological implications of circHIF1A in breast cancer in vivo

**Fig. 7:**
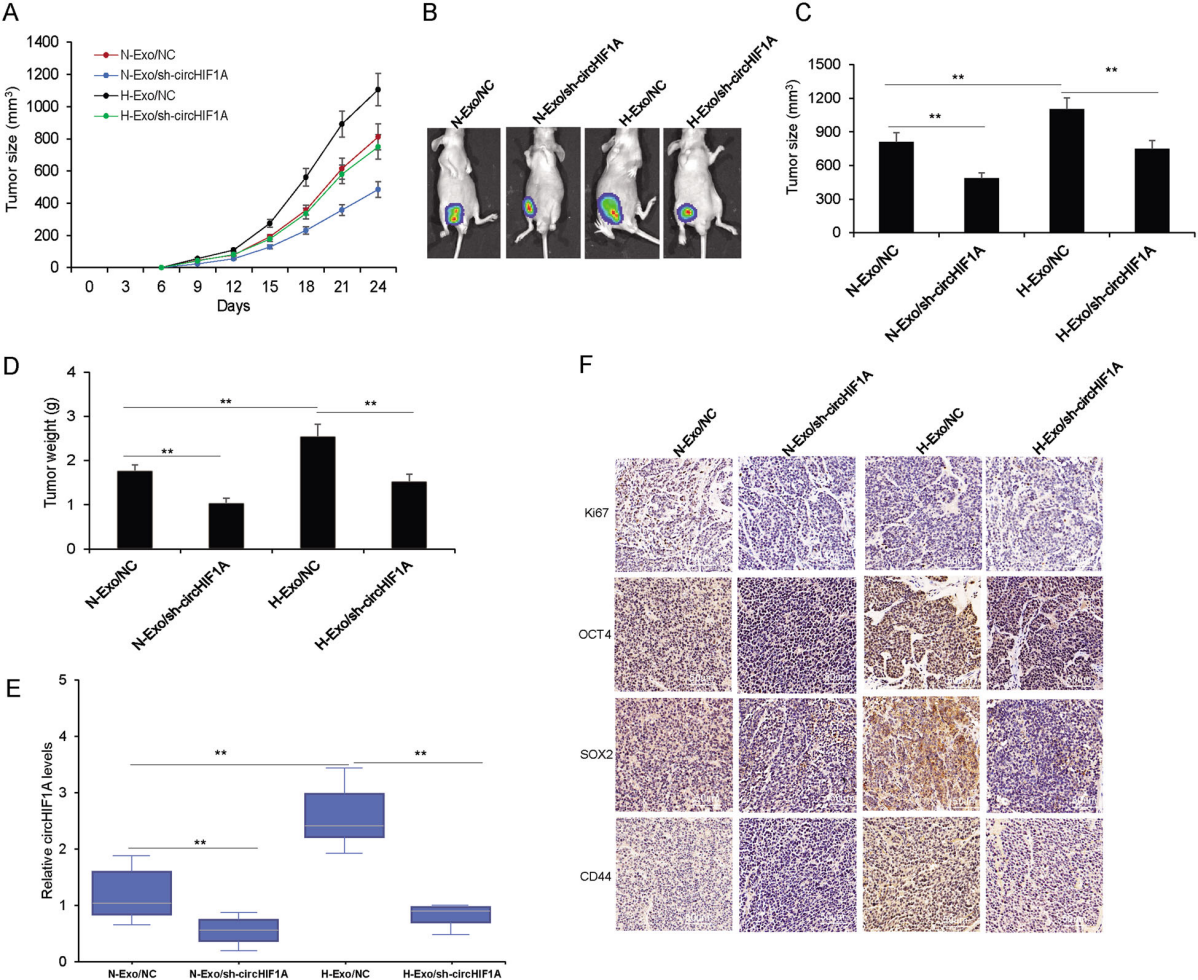
Biological implications of circHIF1A in breast cancer in vivo. **A** The growth curve of breast cancer orthotopic models with circHIF1A downregulation. The breast cancer cells were used to establish the models in BALB/C nude mice and hypoxia exosomes with circHIF1A shRNA or the controls were injected into the nude mice. **B** This represents tumor images in vivo experiment. **C** The average tumor size of tumors after tumor excision. **E** The average tumor weight of models. **E** CircHIF1A levels in the blood of the mice. **F** Immunohistochemistry for stem cell marker CD44, OCT4, SOX2, ALDHA1, and Ki67 in the mice tumor tissues. *p < 0.05, **p < 0.01.

To further confirm that circHIF1A could promote breast cancer stemness in vivo, we established a breast cancer model in BALB/C nude mice. The tumors were established using MDA-MB-231 cells with hypoxic and normoxic CAFs exosomes or hypoxic and normoxic CAFs exosomes with circHIF1A downregulation (Fig. 7A). The growth curve was shown in Fig. 7B. The average tumor size of sh-circHIF1A was much smaller than the control group (Fig. 7C). Tumor with H-exo/ sh-circHIF1A group had a lighter weight than the control group (Fig. 7D). It was also indicated that exosome circHIF1A levels in the serum of mice were lower in the mice than it in the mice with exo/sh-circHIF1A treatment (Fig. 7E). In addition, the resected tumors were made into paraffin-embedded sections, followed by the detection of CD44, SOX2, ALDHA1, and Ki67. H-exo/sh-circHIF1A mice showed lower levels of CD44, SOX2, ALDHA1, and Ki67 than the control ones (Fig. 7F). The results demonstrate that circHIF1A plays an important role in promoting breast cancer stemness in vivo.

## Discussion

Fibroblasts can be activated to CAFs which function as a support for cancer progression including metabolism, metastasis, proliferation, anti-apoptosis, angiogenesis, and therapy resistance^3–5^. Hypoxia is one of the known tumor microenvironmental conditions in regulating fibroblasts activation, however, hypoxic exosomes from tumor stroma are not elucidated. In this study, through circRNA array and whole genome array on the hypoxic and normoxic CAFs, some upregulated and downregulated circRNAs were verified. By further investigation, it was demonstrated that circHIF1A in hypoxic CAFs exosomes could regulate breast cancer cell stemness via miR-580-5p/CD44.

Exosomes secreted from donor cells and then entered into the target cells through fusing with the plasma membrane or through endocytosis, affecting biological events and cellular activity^5,6^. Previous studies of breast cancer have characterized exosomes originating from different types of donor cells like CAFs and revealed their effects on cell growth, metastasis, and drug resistance, indicating material exchange between donor and receipt cells^1–3^. It has been suggested that exosomes communicate with neighboring or distant cells by the horizontal transfer of their cargo molecules to recipient cells, thereby influencing cancer progression and metastasis^6–8^. In this study, we found that CAFs secreted more exosomes in hypoxia than it in normoxia, which indicates that hypoxia could stimulate CAFs to produce more exosomes release.

Among these cargo molecules, RNAs, particularly noncoding RNAs such as lncRNAs, miRNAs, and circRNAs, have been identified as being specifically expressed under different physiological and pathological conditions^4^. In this study, the divergent exosomal circRNAs between hypoxic and normoxic MDA-MB-231 exosomes were compared through circRNA array and whole-genome array. The data showed similar patterns of circRNA distribution in the exosomes from hypoxic cells compared with normoxic cells. Hypoxia altered the cell circRNA expression pattern of exosomes. The exosomes in hypoxic exosomes from CAFs could promote breast cancer stemness.

CircRNA acts as a regulator during carcinogenesis and cancer progression, which is not fully elucidated, but many researchers have stated that circRNAs can function as ceRNAs in tumor biology^9,10^. With the development of our knowledge of circRNAs, many researchers have recognized that circRNAs do not only act as miRNA sponges, and some circRNAs can sponge trans-acting elements to promote or block the transcription of parental genes^9,10^. There is no previous study that analyzed the hypoxic exosomal circRNA in breast cancer. Our study showed that circHIF1A acted as a sponge absorbing miR-580-5p to modulate breast cancer stem cell properties. Further investigation showed that CD44 was a target gene of miR-580-5p.

Hypoxia is related to exosomes non-coding RNA. Previous studies reported that hypoxic BMSC-derived exosomal miRNAs promote metastasis of lung cancer cells^26^; hypoxic cancer cell exosomal circ0048117 facilitates M2 macrophage polarization in esophageal squamous cell carcinoma^27^; hypoxia-induced exosomal circRNA promotes metastasis of colorectal cancer via targeting GEF-H1/RhoA axis^28^; hypoxia-associated circDENND2A promotes glioma aggressiveness by sponging miR-625-5p^29^. Our data demonstrated that circHIF1A from hypoxic CAFs exosomes promoted breast cancer stemness by regulating miR-580-5p targeting CD44 expression.

**Fig. 8:**
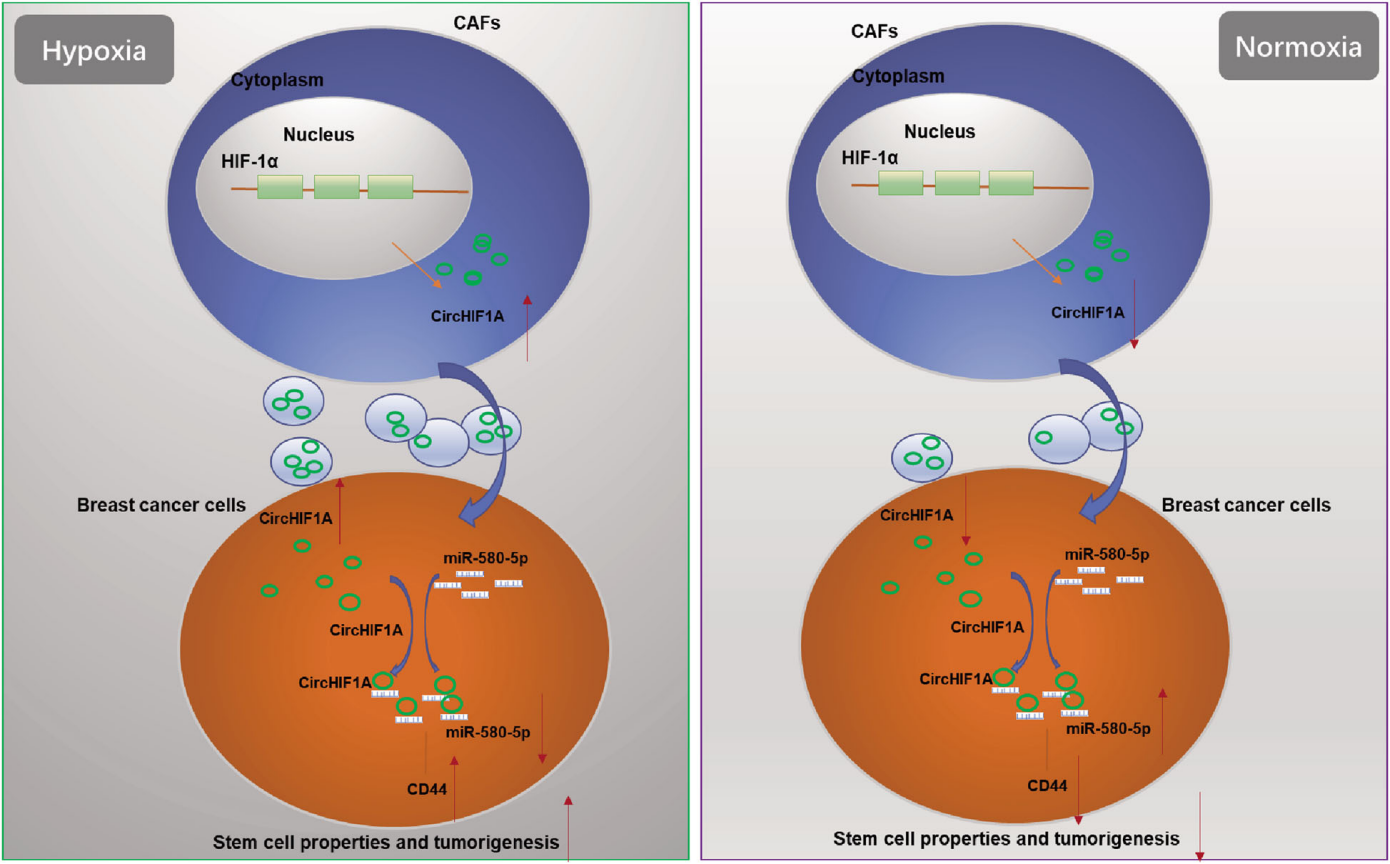
Schematic of the biological roles of circHIF1A from CAFs exosomes in breast cancer. In hypoxia, CAFs derived exosomal circHIF1A could bind to miR-580-5p as a miRNA sponge, promoting breast cancer stem cell properties by upregulating CD44. In normoxia, the above phenotype was suppressed.

In this study, by screening the circRNAs of the exosomes from hypoxic and normoxic tumor stroma cells, circHIF1A was one of the significantly upregulated circRNA in the exosomes from hypoxic CAFs. Further studies were carried out to demonstrate that circHIF1A from hypoxic CAFs exosomes regulated cancer cell stemness by sponging miR-580-5p which targeting CD44 expression in breast cancer, however, in normoxia, low levels of circHIF1A in CAFs exosomes maintains the low stem cell population (Fig. 8). The study for the first time demonstrated that the role of circHIF1A from hypoxic CAFs exosomes in breast cancer and may be a target for breast cancer therapy.

## Supplementary information

Supplementary material Table S1

Figure S2

Figure S3

Supplementary material Figure legends

Figure S1

## References

[1] Gascard P, Tlsty TD (2016). Carcinoma-associated fibroblasts: orchestrating the composition of malignancy. Genes Dev..

[2] Santi A, Kugeratski FG, Zanivan S (2018). Cancer associated fibroblasts: the architects of stroma remodeling. Proteomics.

[3] Prakash J (2016). Cancer-associated fibroblasts: perspectives in cancer therapy. Trends Cancer.

[4] Yang X, Li Y, Zou L, Zhu Z (2019). Role of exosomes in crosstalk between cancer-associated fibroblasts and cancer cells. Front. Oncol..

[5] Whiteside TL (2018). Exosome and mesenchymal stem cell cross-talk in the tumor microenvironment. Semin. Immunol..

[6] Fu H, Yang H, Zhang X, Xu W (2016). The emerging roles of exosomes in tumor-stroma interaction. J. Cancer Res. Clin. Oncol..

[7] Alimirzaie S, Bagherzadeh M, Akbari MR (2019). Liquid biopsy in breast cancer: a comprehensive review. Clin. Genet..

[8] Klinge CM (2018). Non-coding RNAs in breast cancer: intracellular and intercellular communication. Noncoding RNA.

[9] Fanale D, Taverna S, Russo A, Bazan V (2018). Circular RNA in exosomes. Adv. Exp. Med. Biol..

[10] Li Y (2015). Circular RNA is enriched and stable in exosomes: a promising biomarker for cancer diagnosis. Cell Res..

[11] Kristensen LS, Hansen TB, Venø MT, Kjems J (2018). Circular RNAs in cancer: opportunities and challenges in the field. Oncogene.

[12] Hou J (2018). Circular RNAs and exosomes in cancer: a mysterious connection. Clin. Transl. Oncol..

[13] Hu JL (2019). CAFs secreted exosomes promote metastasis and chemotherapy resistance by enhancing cell stemness and epithelial-mesenchymal transition in colorectal cancer. Mol. Cancer.

[14] Wang H (2020). MicroRNA-181d-5p-containing exosomes derived from CAFs promote EMT by regulating CDX2/HOXA5 in breast cancer. Mol. Ther. Nucleic Acids.

[15] Li YY (2018). Cancer-associated fibroblasts contribute to oral cancer cells proliferation and metastasis via exosome-mediated paracrine miR-34a-5p. EBioMedicine.

[16] Ren J (2018). Carcinoma-associated fibroblasts promote the stemness and chemoresistance of colorectal cancer by transferring exosomal lncRNA H19. Theranostics.

[17] Qin X (2019). Exosomal miR-196a derived from cancer-associated fibroblasts confers cisplatin resistance in head and neck cancer through targeting CDKN1B and ING5. Genome Biol..

[18] Choudhry H, Harris AL (2018). Advances in hypoxia-inducible factor biology. Cell Metab..

[19] Peng X (2020). The interplay between HIF-1α and noncoding RNAs in cancer. J. Exp. Clin. Cancer Res..

[20] Cao L (2020). Circular RNA circRNF20 promotes breast cancer tumorigenesis and Warburg effect through miR-487a/HIF-1α/HK2. Cell Death Dis..

[21] Shao C (2018). Role of hypoxia-induced exosomes in tumor biology. Mol. Cancer.

[22] Deep G, Panigrahi GK (2015). Hypoxia-induced signaling promotes prostate cancer progression: exosomes role as messenger of hypoxic response in tumor microenvironment. Crit. Rev. Oncog..

[23] Maia J, Caja S, Strano Moraes MC, Couto N, Costa-Silva B (2018). Exosome-based cell-cell communication in the tumor microenvironment. Front. Cell Dev. Biol..

[24] Ramteke A (2015). Exosomes secreted under hypoxia enhance invasiveness and stemness of prostate cancer cells by targeting adherens junction molecules. Mol. Carcinog..

[25] King HW, Michael MZ, Gleadle JM (2012). Hypoxic enhancement of exosome release by breast cancer cells. BMC Cancer.

[26] Zhang X (2019). Hypoxic BMSC-derived exosomal miRNAs promote metastasis of lung cancer cells via STAT3-induced EMT. Mol. Cancer.

[27] Lu Q (2020). Hypoxic tumor-derived exosomal circ0048117 facilitates M2 macrophage polarization acting as miR-140 sponge in esophageal squamous cell carcinoma. Onco Targets Ther..

[28] Yang H (2020). Hypoxia induced exosomal circRNA promotes metastasis of colorectal cancer via targeting GEF-H1/RhoA axis. Theranostics.

[29] Su H, Zou D, Sun Y, Dai Y (2019). Hypoxia-associated circDENND2A promotes glioma aggressiveness by sponging miR-625-5p. Cell Mol. Biol. Lett..

