# Supplementary material for: Carcinoma-associated fibroblasts derived exosomes modulate breast cancer cell stemness through exonic circHIF1A by miR-580-5p in hypoxic stress
Source: Cell Death Discov. 2021 Jun 12;7:141. doi: 10.1038/s41420-021-00506-z (PMC8197761; doi:10.1038/s41420-021-00506-z)
Supplement: Supplementary file 1 — Supplementary material Table S1 [file 41420_2021_506_MOESM1_ESM.docx]

**Additional file 1: Table S1**

**Table 1. Major primer sequences in this study**

| Primer | Sequences (5′-3′) |
| --- | --- |
| HIF-1α | FP: AGTGTACCCTAACTAGCCGAGGAA  RP: CTGAGGTTGGTTACTGTTGGTATCA |
|  |  |
| OCT4 | FP: CTTGAATCCCGAATGGAAAGGG  RP: GTGTATATCCCAGGGTGATCCTC |
| SOX2 | FP: TTTGTCGGAGACGGAGAAGC  RP: TAACTGTCCATGCGCTGGTT |
| ALDH1 | FP: GCACGCCAGACTTACCTGTC  RP: CCTCCTCAGTTGCAGGATTAAAG |
| CD44 | FP: TTTGCATTGCAGTCAACAGTC  RP: GTTACACCCCAATCTTCATGTCCAC |
| NANOG | FP: TTTGTGGGCCTGAAGAAAACT  RP: AGGGCTGTCCTGAATAAGCAG |
| GAPDH | FP: ACCACAGTCCATGCCATCAC  RP: CCACCACCCTGTTGCTGTAG |
| U6 | FP: CTCGCTTCGGCAGCACA  RP: AACGCTTCACGAATTTGCGT |
| circ_0032138 | FP: AGGACAGTACAGGATGCTTGCC  RP: ATATCCCATCAATTCGGTAATTCTC |

FP: forward primer; RP: reverse primer
