# Supplementary figures and images for: Carcinoma-associated fibroblasts derived exosomes modulate breast cancer cell stemness through exonic circHIF1A by miR-580-5p in hypoxic stress
Source: Cell Death Discov. 2021 Jun 12;7:141. doi: 10.1038/s41420-021-00506-z (PMC8197761; doi:10.1038/s41420-021-00506-z)

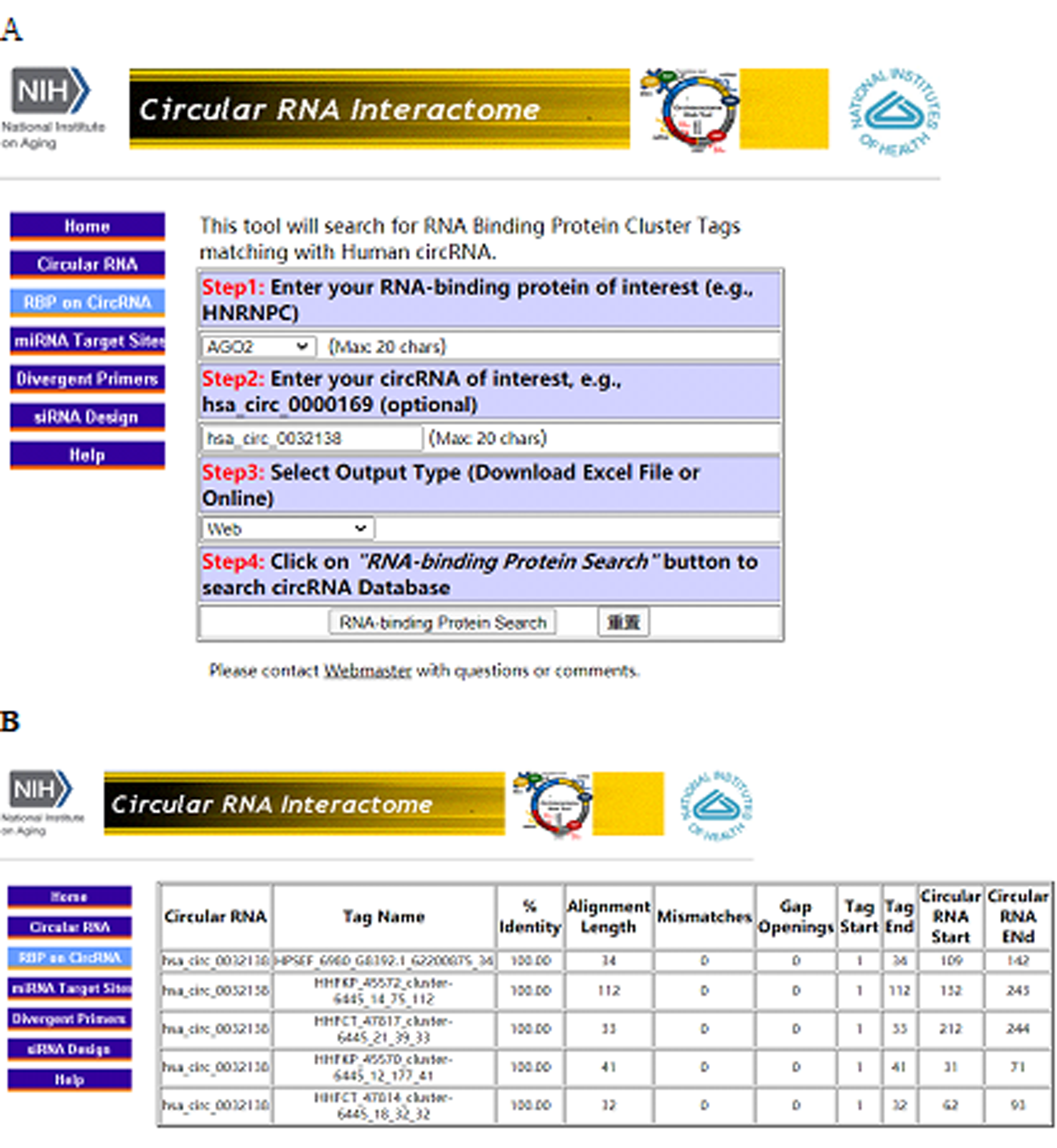

Supplement: Supplementary file 2 — Figure S2 [file 41420_2021_506_MOESM2_ESM.tif]

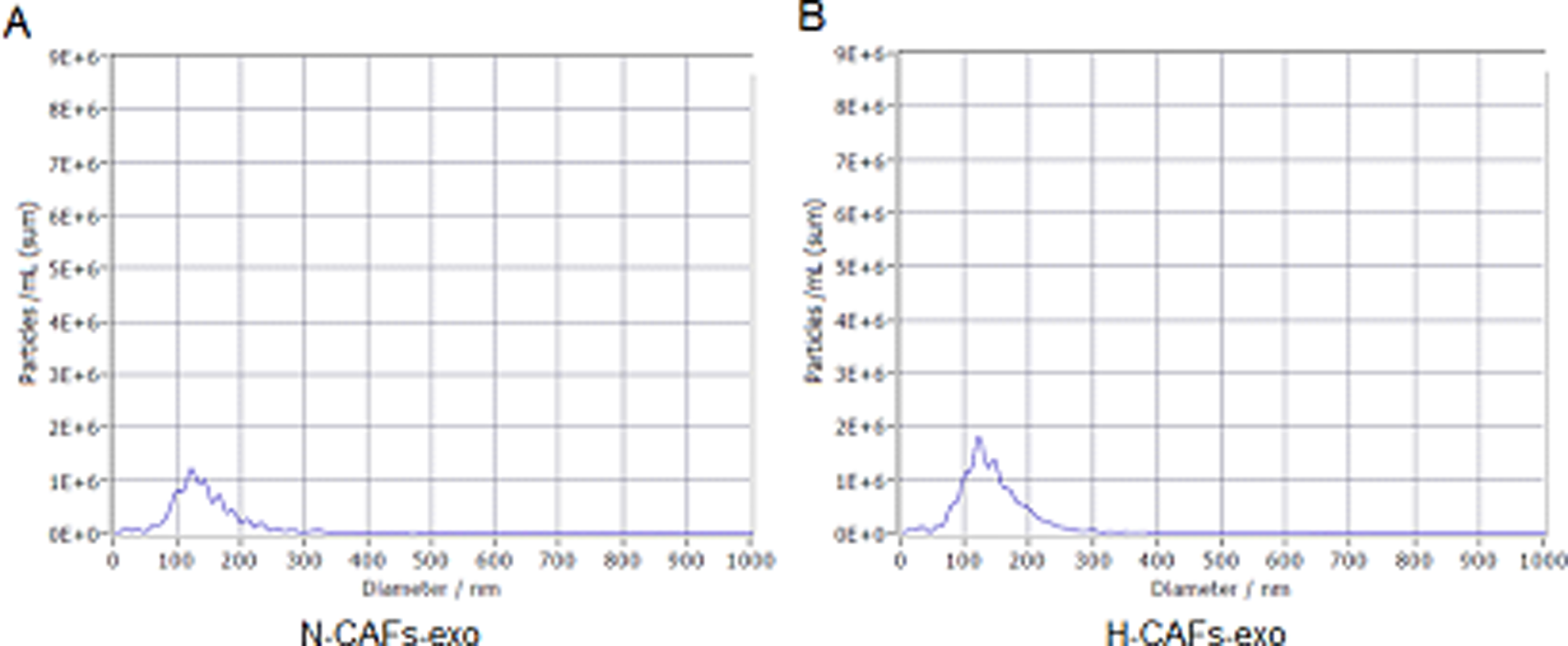

Supplement: Supplementary file 3 — Figure S3 [file 41420_2021_506_MOESM3_ESM.tif]

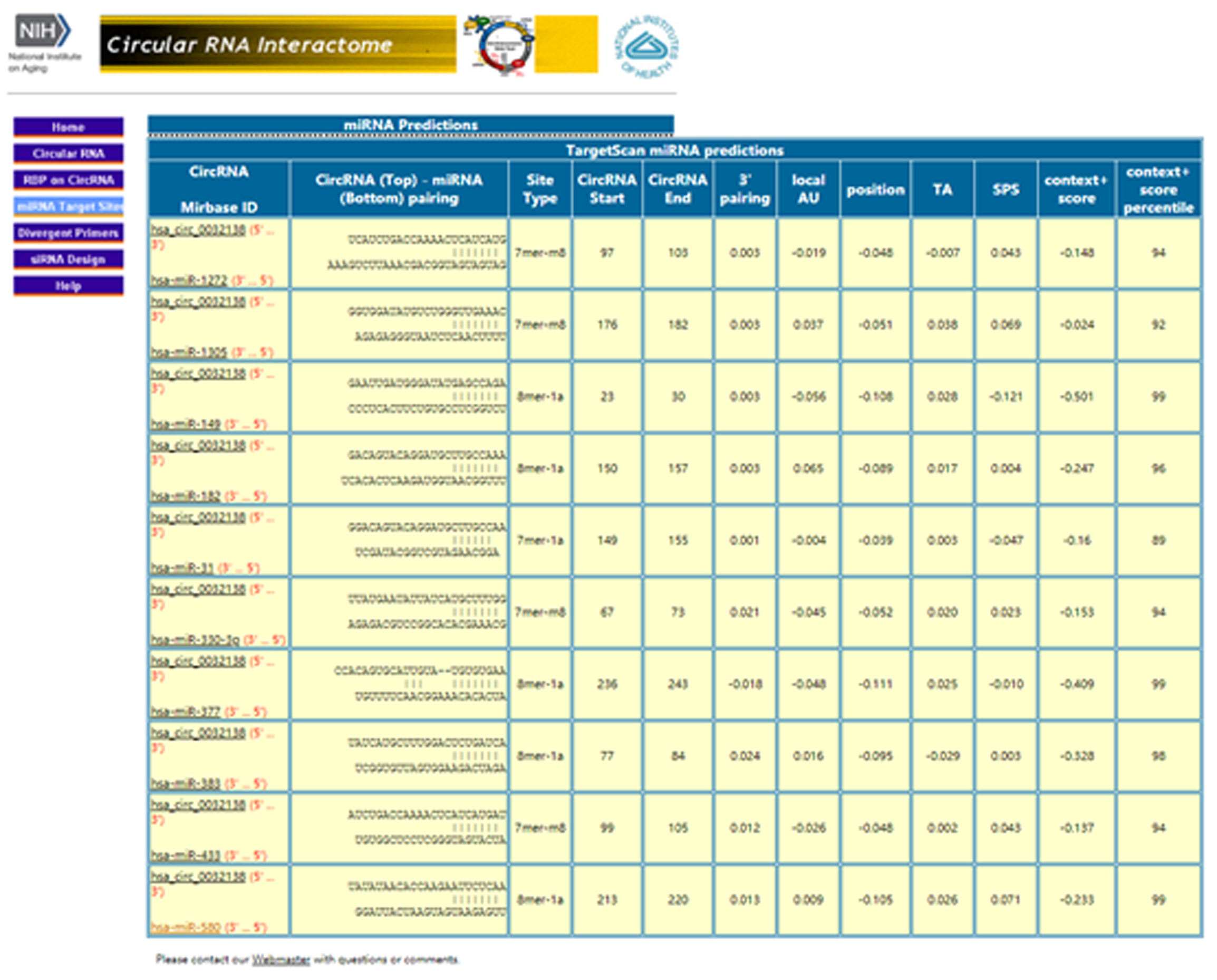

Supplement: Supplementary file 5 — Figure S1 [file 41420_2021_506_MOESM5_ESM.tif]
