# Supplementary material for: Carcinoma-associated fibroblasts derived exosomes modulate breast cancer cell stemness through exonic circHIF1A by miR-580-5p in hypoxic stress
Source: Cell Death Discov. 2021 Jun 12;7:141. doi: 10.1038/s41420-021-00506-z (PMC8197761; doi:10.1038/s41420-021-00506-z)
Supplement: Supplementary file 4 — Supplementary material Figure legends [file 41420_2021_506_MOESM4_ESM.docx]

**Supplementary material Figure legends**

**Figure S1: The interaction of circHIF1A and miRNA were predicted.** https://circinteractome.nia.nih.gov/api/v2/mirnasearch?circular_rna_query=hsa_circ_0032138&mirna_query=&submit=miRNA+Target+Search

**Figure S2**. **The AGO2 might bind to circHIF1A (hsa_circ_0032138).** It was predicted with circInteractome database (https://circinteractome.nia.nih.gov/index.html). **A** AGO2 was selected for finding the RNA binding protein of hsa_circ_0032138. **B** the searching result.

**Figure S3. NTA analysis of exosomes from CAFs with normoxia and hypoxia treatment.** CAFs were cultured under normoxia or hypoxia for 24h and then the medium was collected for exosomes isolation. A: Exosome particles from CAFs under normoxia. B: Exosome particles from CAFs under hypoxia.
